# Seroprevalence of *Alphavirus* Antibodies in a Cross-Sectional Study in Southwestern Tanzania Suggests Endemic Circulation of Chikungunya

**DOI:** 10.1371/journal.pntd.0002979

**Published:** 2014-07-31

**Authors:** Nina Weller, Petra Clowes, Gerhard Dobler, Elmar Saathoff, Inge Kroidl, Nyanda Elias Ntinginya, Leonard Maboko, Thomas Löscher, Michael Hoelscher, Norbert Heinrich

**Affiliations:** 1 Division of Infectious Diseases and Tropical Medicine, Medical Centre of the University of Munich (LMU), Munich, Germany; 2 National Institute for Medical Research -Mbeya Medical Research Center, Mbeya, Tanzania; 3 Bundeswehr Institute of Microbiology, Munich, Germany; 4 German Center for Infection Research (DZIF), partner site Munich, Munich, Germany; University of Texas Medical Branch, United States of America

## Abstract

**Background:**

To date, *Alphavirus* infections and their most prominent member, chikungunya fever, a viral disease which first became apparent in Tanzania in 1953, have been very little investigated in regions without epidemic occurrence. Few data exist on burden of disease and socio-economic and environmental covariates disposing to infection.

**Methods:**

A cross-sectional seroprevalence study was undertaken in 1,215 persons from Mbeya region, South-Western Tanzania, to determine the seroprevalence of anti-*Alphavirus* IgG antibodies, and to investigate associated risk factors.

**Results:**

18% of 1,215 samples were positive for *Alphavirus* IgG. Seropositivity was associated with participant age, low to intermediate elevation, flat terrain and with IgG positivity for Rift Valley fever, *Flaviviridae*, and rickettsiae of the spotted fever group. When comparing the geographical distribution of *Alphavirus* seropositivity to that of Rift Valley fever, it was obvious that *Alphaviruses* had spread more widely throughout the study area, while Rift Valley fever was concentrated along the shore of Lake Malawi.

**Conclusion:**

*Alphavirus* infections may contribute significantly to the febrile disease burden in the study area, and are associated with several arthropod-borne infections. Their spread seems only limited by factors affecting mosquitoes, and seems less restricted than that of Rift Valley fever.

## Author Summary

The origin of febrile disease is often difficult to diagnose. In tropical countries, viral infections that are transmitted by arthropods include, among others, *Alphavirus* infections (e.g. chikungunya fever), dengue, West Nile, Yellow Fever and Rift Valley fever. In malaria endemic areas, these diseases are often mis-diagnosed and treated as malaria. Our study examined serum samples from 1,215 participants of a population survey from the Mbeya region, south-western Tanzania, for antibodies against *Alphaviruses* of the Semliki forest group as a sign of past infection. We found 18% of study participants positive, a surprisingly high number which points to a hitherto undetected circulation of *Alphaviruses* in the region. Among examined risk factors, even terrain, low to moderate elevation and participant age were associated with antibody positivity. Comparison with the distribution of Rift Valley fever seropositivity showed that *Alphaviruses* are more widely distributed throughout the study area, while Rift Valley fever seems to occur in a limited area close to Lake Malawi only.

## Introduction

*Alphaviruses* form a genus in the family *Togaviridae*. About 40 different viruses including type and sub-type viruses are members of the genus. Among them are major human pathogens such as chikungunya virus (CHIKV) and viruses of veterinary importance, e.g. Venezuelan equine encephalitis virus. The currently most important *Alphavirus* of human pathogenicity is CHIKV, which causes significant morbidity and economic losses [1]. Although it has been described and isolated first in 1953 from a febrile person in Tanzania, East Africa [2], currently only few data on the distribution and medical importance of CHIKV and other *Alphaviruses* in Africa are available.

Since the 1960s, especially CHIKV was repeatedly isolated throughout African and Asian countries [3], and small outbreaks were frequently reported. The virus gained notoriety, when in the years 2004–2007 an outbreak was noticed of so far unknown dimension. Starting in Kenya, a severe epidemic hit the islands of the Indian Ocean in 2005/2006, with nearly 280.000 people infected on the island of La Reunion alone [1], [4], [5]. Transmission to the Indian sub-continent resulted in chikungunya fever in an estimated 1.3 million people [6]. The enormous scientific interest in this outbreak led to several new findings concerning viral molecular biology and ecology [3], [7]–[9]. Investigations regarding the climatic conditions before the outbreak revealed unusual warm and dry conditions along the Kenyan coast in 2004 [10], [11]. Infrequent replenishment of domestic water stores due to these dry conditions may have facilitated the transmission of the virus.

Despite this increased research interest, the role of CHIKV as well as other *Alphaviruses* in endemic regions, especially in sub-Saharan Africa, remains unclear. Recent studies concentrated mainly on areas of the latest CHIKV pandemic. The disease burden and the epidemiology in local populations not affected by the devastating outbreak in 2004–2007 is largely unknown. In a small study in Guinea, arboviruses as causative agent for febrile disease were identified by neutralization assays in 63% of 47 patients [12]. 17% of these had acute CHIKV infections. In a clinical study conducted in Northern Tanzania with 870 febrile patients, PCR-confirmed acute CHIKV infections were diagnosed in 7.9% of all cases [13]. However, surveillance of other *Alphaviruses* is even less developed as most of these studies are targeting CHIKV using PCR. A serosurvey in rural Kenya revealed a seropositivity prevalence of 34% for anti-*Alphavirus* IgG, which was not associated with age, indicating frequently occurring smaller epidemics rather than endemic cycling [14]. Although CHIKV is expected as the main pathogen, other *Alphaviruses* cannot be excluded since a broadly cross-reactive ELISA was used.

With the recent outbreak of CHIKV in Italy, and detection of autochthonous transmission in southern France, it is clear that *Alphaviruses* and especially CHIKV have the potential to become endemic in areas in Europe where *Aedes albopictus* is already established [15], [16].

In this study we aimed to assess the epidemiological patterns of *Alphavirus* infections in the Mbeya Region in Tanzania, by measuring seroprevalence in 1215 individuals participating in an epidemiological survey in the Region. This region was not affected by the 2004–2007 outbreak, and diagnosis or laboratory verification of acute chikungunya fever or other *Alphavirus* infection does not occur locally. The survey gave us the opportunity to study the role of this pathogen genus and its dependence on certain social and ecological factors in an endemic transmission cycle in a typical local setting.

## Materials and Methods

### Ethics statement

Both EMINI and this sub-study were approved by local and national Tanzanian ethics committees. Each EMINI participant had provided written informed consent before enrolment, with parents consenting for their children.

### Study population and the EMINI survey

The EMINI population survey had the objective to create the infrastructure to **E**valuate and **M**onitor the **I**mpact of **N**ew **I**nterventions in the Mbeya Region of south-western Tanzania. Financed by the European Union over five years (2006 to 2011), the strengthening of the local health infrastructure and the establishment of a cohort which could be followed up on an annual basis created a platform on which the impact of improved health care infrastructure and new interventions could be monitored and evaluated. Embedded into the EMINI project were several focused studies such as this sub-study, which determined seroprevalences for a number of tropical arthropod-borne diseases.

In preparation of the EMINI survey, a census of the entire population in nine geographically distinct and ecologically different sites of the Mbeya Region was carried out. Study sites were selected to reflect the wide range of different conditions within the region in terms of elevation, population density and development (urban versus rural). Basic information regarding the households and their inhabitants was collected and all household positions were recorded with handheld GPS receivers. Ten percent of the surveyed households were then chosen by geographically stratified random selection for inclusion into the EMINI survey, to obtain a representative sample of the population from each site. The resulting EMINI cohort included all consenting participants of 4,283 households. Over the following five years annual visits at the same time of the year were conducted, during which structured interviews with all household members were performed, and blood, urine and stool specimens collected.

For this sub-study, we stratified the 17,872 participants, who had provided a blood sample during the second EMINI survey in 2007/2008, by age, gender, altitude of residence and ownership of domestic mammals. To be able to assess factors of interest that were identified in the literature but might have been underrepresented in the study population, we employed disproportionate random sampling with equal participant numbers for each stratum to identify 1.226 samples from participants above the age of 5 years to be tested for IgG antibodies against *Alphaviruses* and other tropical arthropod-borne diseases.

### Socio-economic status

To characterize the socio-economic situation of each household, the head of each household was asked for the following information during each annual EMINI visit: Presence/absence of different items in the household (clock or watch, radio, television, mobile telephone, refrigerator, hand cart, bicycle, motor cycle, car, savings account), sources of energy and drinking water, materials used to build the main house, number of persons per room in the household and availability and type of latrine used. Based on the provided information, a socio-economic-status (SES) score was established, using a modified method originally proposed by Filmer and Pritchett which has frequently been employed to characterize the SES of people living in developing countries [17]–[19].

### Environmental data

Population- and livestock-densities were calculated using data and household positions collected during the initial population census. Elevation data were retrieved from the NASA Shuttle Radar Topography Mission (SRTM) global digital elevation model, version 2.1 [20], [21].

Land surface temperature (LST) and vegetation cover (EVI =  enhanced vegetation index) data for the years 2003 to 2008 were retrieved from NASA's Moderate-resolution Imaging Spectroradiometer (MODIS) Terra mission which “are distributed by the Land Processes Distributed Active Archive Center (LP DAAC), located at the U.S. Geological Survey (USGS) Earth Resources Observation and Science (EROS) Center (lpdaac.usgs.gov)” [22]. These data were used to produce long-term averages of day and night LST and EVI.

Population-, household-, and livestock-densities, LST, EVI, and elevation data were averaged for a buffer area within 1000 meter radius around each household in order to characterize the ecological situation around the household. This approach was preferred to using the respective spot values at the household position, because spot data are more prone to random error than averages for a wider area.

### Serology

Detection of anti-*Alphavirus* IgG, anti-Yellow fever virus IgG, anti-dengue 1–4 virus IgG, and anti-West Nile virus IgG on bio-banked samples were performed as described previously for Rift Valley fever virus (RVfV) IgG [23]. A commercially available biochip (Euroimmun, Lübeck, Germany), containing infected and non-infected Vero E6 cells or only non-infected Vero E6 cells (negative control), was used for indirect immunfluorescence testing (IIFT), following a standard protocol. All serum samples were heat-inactivated and diluted tenfold prior to testing. Further dilutions of positive sera were carried out in the range of 1∶20 to 1∶640. A rabbit anti-human IgG FITC-labelled antibody (DAKO, Hamburg, Germany) served as conjugate. To decrease the known subjectivity of reading IIFT results to the best objective level, fluorescence microscopy was carried out independently by two experienced observers. In case of discrepancies (positive vs. negative; difference >1 titer step) the testing was repeated. A sample was classified as positive, if at a screening dilution of 1∶20 a typical fine granular cytoplasmatic fluorescence was detected in around 20% of the cells on the positive field of the biochip with a typical location and morphology of infected cells, while no signal was detectable in the negative field. IIFT was repeated in case of indeterminate results, i.e. in cases where samples differed clearly from the negative control but did not match the criterion “positive”. Ultimately, 1,215 definitive results were available from the selected samples.

### Testing for *P. falciparum* malaria

Fresh EDTA-blood was used for malaria testing using a rapid test (ICT, South Africa) for each participant.

### Data analysis

Stata statistics software (version 12, Statacorp, College Station, TX, USA) was used for all statistical analyses, and Manifold System 8.0 Professional Edition (Manifold Net Ltd, Carson City, NV) was used for processing of geographical data and to produce maps. In order to identify possible risk factors for anti-*Alphavirus* IgG positivity, we analysed seropositivity as the binary outcome in uni- and multi-variable Poisson regression models with robust (or Huber-White) variance estimates adjusted for household clustering [24], [25]. Initial uni-variable models for all factors that we deemed as possibly related to CHIKV infection were used to identify variables with a uni-variable p-value < = 0.1 for further multi-variable evaluation. Stepwise backward and forward regression, the Akaike and Bayes information criterion and various assessments of model-fit were used to identify the best multi-variable model, where only variables with a multi-variable p-value <0.1 were retained.

Associations of anti-*Alphavirus* IgG positivity with other diseases were assessed in uni-variable Poisson regression models with anti-*Alphavirus* IgG as the binary outcome, and the respective disease as the only predictor variable. In addition we ran the same models adjusted for those risk factors that had been retained in the above described multi-variable models regarding risk factors for anti-*Alphavirus* IgG positivity.

In the analysis, positivity for at least one of dengue, West Nile and Yellow fever antibodies, was categorized as “*Flavivirus* IgG” positive.

## Results

**Figure 1:**
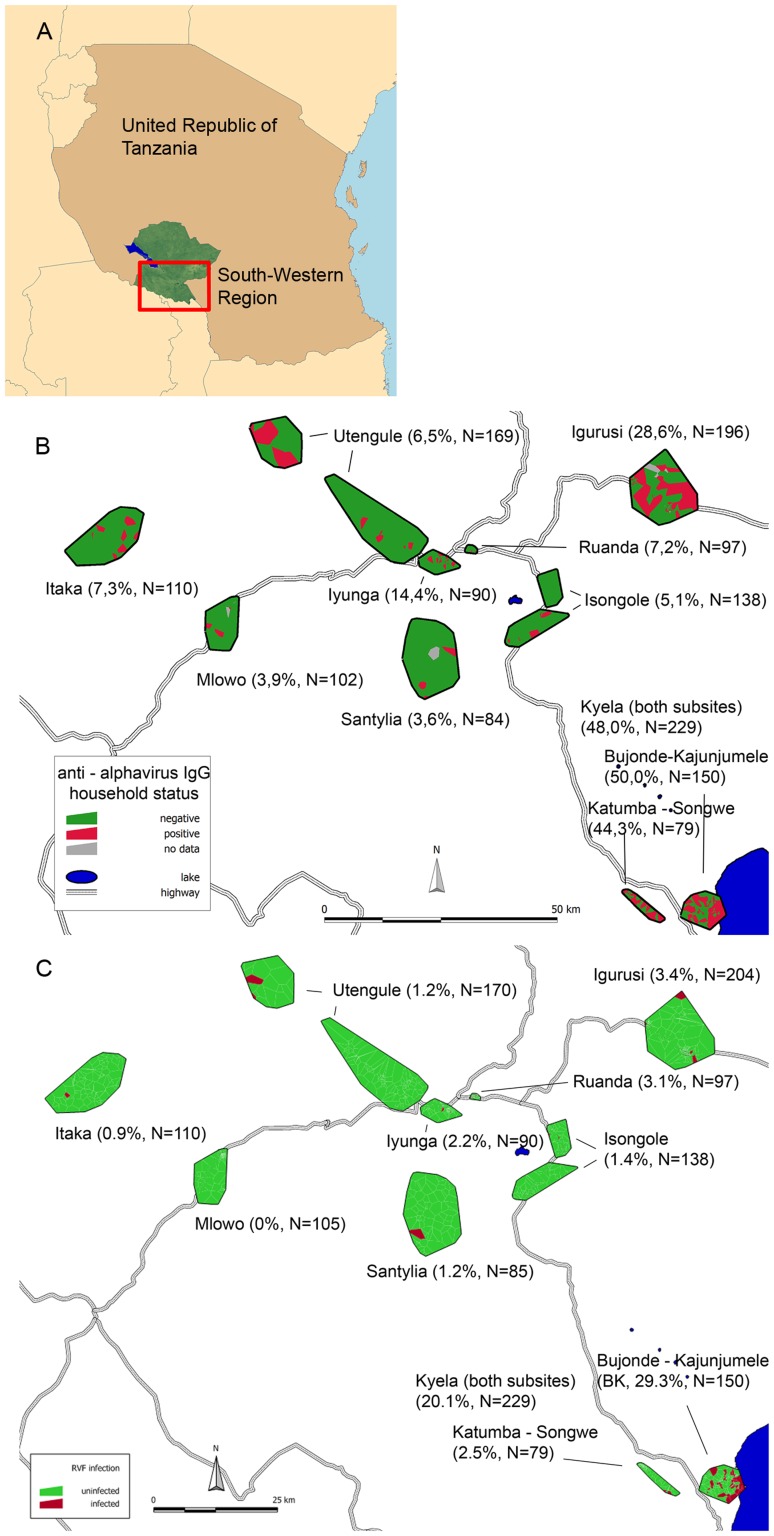
Location of Households with positive participants for anti-*Alphavirus* IgG and Rift Valley fever virus IgG. Localisation of the study area in Tanzania (A). Location of households with (B) *Alphavirus* IgG-positive and (C) Rift Valley fever virus IgG-positive participants displayed as Voronoi polygons, with every polygon representing one household. Percent IgG-positives and total N examined in the site are displayed with site name. Households with one or more individuals positive for *Alphavirus* IgG are marked in red, all others in green. For Kyela site, both subsites Bujonde-Kajunjumele and Katumba-Songwe are displayed. Map created by use of Manifold System 8.0 software. (C) reproduced from [23] under creative commons license.

219 of 1,215 (18.0%) samples reacted positive for anti-*Alphavirus* IgG. The estimated overall population prevalence, predicted from our stratified sample by direct extrapolation, is 11.8% for the population of our 9 sites (fig. 1).

Seropositivity increased with participant age, both in uni- and in multi-variable analysis (table 1; prevalence ratio (PR) for a 10-year increase in multi-variable analysis: 1.26, 95% confidence interval (CI) 1.20 to 1.32, p<0.001). Gender was not significantly associated with seropositivity.

**Table 1:** Association of anti-*Alphavirus* IgG status with environmental and socio-economic factors.

				uni-variable^1^			multi-variable^2^		
Covariate	stratum	N	% pos.	PR	95% conf.int.	p-val	PR	95%conf.int.	p-val
**Age**									
	per 10 years	1215		1.24	(1.18 to 1.30)	<0.001	1.26	(1.20 to 1.32)	<0.001
**Slope**									
	per degree	1215		0.58	(0.50 to 0.66)	<0.001	0.86	(0.77 to 0.95)	0.004
E**levation (m)**									
	479.1-	122	50.0	1	-	-	1	-	-
	487.5-	122	43.4	0.87	(0.66 to 1.14)	0.317	1.09	(0.85 to 1.41)	0.504
	973.7-	118	33.9	0.68	(0.49 to 0.94)	0.019	0.73	(0.54 to 0.98)	0.038
	1197.8-	120	15.8	0.32	(0.20 to 0.51)	<0.001	0.41	(0.25 to 0.66)	<0.001
	1290.9-	123	4.9	0.10	(0.04 to 0.22)	<0.001	0.16	(0.07 to 0.37)	<0.001
	1491.4-	120	4.2	0.08	(0.03 to 0.20)	<0.001	0.13	(0.05 to 0.34)	<0.001
	1578.0-	122	10.7	0.21	(0.12 to 0.37)	<0.001	0.27	(0.15 to 0.47)	<0.001
	1612.8-	123	9.8	0.20	(0.11 to 0.35)	<0.001	0.23	(0.13 to 0.40)	<0.001
	1724.5-	123	5.7	0.11	(0.05 to 0.24)	<0.001	0.19	(0.08 to 0.43)	<0.001
	2002.8-	122	2.5	0.05	(0.02 to 0.15)	<0.001	0.10	(0.03 to 0.34)	<0.001
**Gender**							*This and the following variables were not included into multi-variable analysis due to lack of multi-variable significance.*		
	female	667	17.1	1	-	-			
	male	540	19.3	1.13	(0.89 to 1.42)	0.318			
	missing data	8	12.5	0.73	(0.12 to 4.61)	0.739			
**SES Rank**									
	per unit	1215		0.90	(0.86 to 0.94)	<0.001			
**Bednet owned**									
	No	692	10.4	1	-	-			
	Yes	523	28.1	2.70	(2.07 to 3.53)	<0.001			
**Frequency of bednet use**									
	Never	694	10.2	1	-	-			
	Sometimes	55	16.4	1.60	(0.84 to 3.05)	0.153			
	Most times	21	23.8	2.33	(1.10 to 4.94)	0.028			
	Always	443	30.0	2.93	(2.25 to 3.84)	<0.001			
	missing data	2	50.0	4.89	(1.20 to 19.87)	0.027			
**Persons/km^2^**									
	per unit	1215		0.92	(0.87 to 0.97)	0.002			
**Enhanced Vegetation Index (Max.)**									
	per 0,1 units	1215		2.03	(1.56 to 2.65)	<0.001			
**Enhanced Vegetation Index (Avg.)**									
	per 0,1 units	1215		2.53	(2.04 to 3.14)	<0.001			
**Enhanced Vegetation Index (Min.)**									
	per 0,1 units	1215		2.49	(1.92 to 3.23)	<0.001			
**Average Land Surface Temperature**									
	per 10°	1215		1.73	(1.02 to 2.93)	0.043			
**Night Land Surface Temperature**									
	per 10°	1215		9.70	(6.91 to 13.60)	<0.001			
**Rainfall**									
	per unit	1215		1.01	(1.01 to 1.01)	<0.001			

Results of Poisson regression models adjusted for household clustering using robust variance estimates.

N =  number of observations; % pos.  =  percent anti-*Alphavirus* IgG positive in stratum; PR  =  Prevalence ratio; 95% conf.int  =  95% confidence interval; SES rank  =  rank (from 0 for lowest to 10 for highest) according to socio-economic score.

1: results of separate models for each of the below covariates.

2: multivariable model including only age, elevation and slope of terrain as covariates.

**Figure 2:**
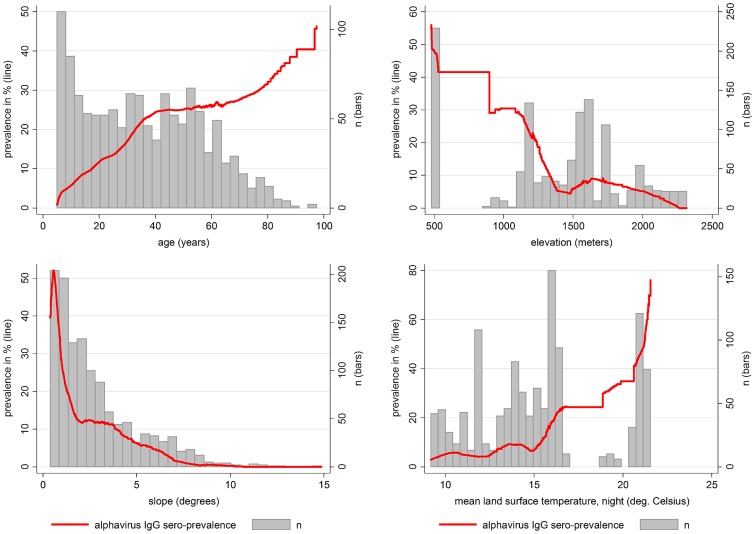
Anti-*Alphavirus* IgG prevalence in association with age, elevation, slope and land surface temperature. Lowess smoothed plots of anti-ChikV IgG-seroposivitivity over age, elevation, slope of terrain, and land surface temperature during the night. Red line: anti-*Alphavirus* seroprevalence. Grey bars: number of observations in stratum.

We found a significant association of anti-*Alphavirus* IgG status with elevation above sea level, with significantly higher seroprevalence in the strata below 1,198 m, both in uni-variable and multi-variable analysis (fig. 2). The median elevation of the Kyela site is 487 m (Interquartile range IQR 483 m–514 m), and that of Igurusi is 1,193 m (IQR 1,156–1,205 m). Not only elevation, but also slope of the terrain was negatively associated with seropositivity in uni- and multi-variable analysis, even when adjusted for age and elevation (PR 0.86 per degree, 95% CI 0.77 to 0.95, p = 0.004), with the highest anti-*Alphavirus* IgG prevalence occurring on terrain with a slope of less than ∼1.6° (fig. 2).

Several social, economic and behavioural factors showed significant association in uni-variable analysis but were rendered non-significant in multi-variable analysis when adjusting for age, elevation and slope of terrain. Factors associated with higher anti-*Alphavirus* IgG prevalence in uni-variable analysis included a lower socio-economic status, lower population density, higher vegetation density and higher land surface temperatures, especially night temperatures. Also, bed net ownership and higher frequency of use, which occurred in areas with higher mosquito burdens, were associated with a higher seropositivity in uni-variable analysis. Anti-*Alphavirus* IgG status was not associated with animal ownership, including cattle, sheep, goats and chicken (data not shown).

Next, we analysed correlations between anti-*Alphavirus* IgG status and other infectious diseases throughout the survey. Uni-variable analyses showed significant positive associations of anti-*Alphavirus* IgG with *P. falciparum* malaria RDT positivity, antibody positivity for spotted fever group rickettsiae (SFG) and Rift Valley fever virus (RVFV) [23], and any of the tested *Flaviviridae* (table 2). In separate models for each of these pathogens, that were adjusted for age, elevation and slope of terrain, a significant positive association was retained for SFG IgG, *Flavivirus* IgG and RVFV IgG, while the association with *P. falciparum* disappeared. A comparison of the spatial distribution of RVFV IgG and anti-*Alphavirus* IgG shows that for both viruses, Kyela site has the highest seroprevalences, but a wider occurrence is seen for anti-*Alphavirus* IgG when compared to RVFV IgG (fig. 1).

**Table 2:** Association of anti-*Alphavirus* IgG status with other infectious diseases.

				uni-variable^1^			adjusted^2^		
Covariate	stratum	N	% pos.	PR	95% conf.int. ^‡^	p	PR	95%conf.int.	p
**SFG Rickettsiae IgG status**									
	neg.	392	9.4	1	-	-	1	-	-
	pos.	823	22.1	2.34	(1.67 to 3.28)	<0.001	1.51	(1.11 to 2.06)	0.008
**Rift Valley fever IgG status**									
	neg.	1151	15.2	1	-	-	1	-	-
	pos.	62	71.0	4.67	(3.78 to 5.76)	<0.001	1.68	(1.25 to 2.25)	0.001
	miss.^3^	2	0.0	ND	-	-	ND	-	-
**Any *Flavivirus* IgG status**									
	neg.	1049	13.3	1	-	-	1	-	-
	pos.	161	49.1	3.68	(2.95 to 4.59)	<0.001	1.34	(1.06 to 1.70)	0.013
	miss.^3^	5	0.0	ND	-	-	ND	-	-
***P. falciparum* status**									
	neg.	1195	17.8	1	-	-	1	-	-
	pos.	18	33.3	1.87	(0.96 to 3.64)	0.065	1.01	(0.53 to 1.94)	0.970
	miss.^3^	2	0.0	ND	-	-	ND	-	-

Results of Poisson regression models adjusted for household clustering using robust variance estimates.

N =  number of observations; % pos.  =  percent anti-*Alphavirus* IgG positive in stratum; PR  =  Prevalence ratio; 95% conf.int  = 95% confidence interval; SFG: spotted fever group rickettsiae; IgG  =  Immunogolbulin G; ND  =  not done.

1: results of separate uni-variable models for each of the above infections.

2: results of separate multi-variable models for each of the above infections adjusted for age, elevation and slope of terrain as covariates.

3: 95% confidence interval and p-value not calculated due to lack of variability of the outcome variable in this stratum.

HIV status was unrelated with individual anti-*Alphavirus* IgG status in uni- and multi-variable analysis (data not shown).

## Discussion

In the current study we present high rates of IgG antibodies against an *Alphavirus*. Cross reactions mainly occur in IFAT between antibodies against CHIKV and other viruses of the Semliki-Forest virus complex of *Alphaviruses*, while cross reactivities against the Venezuelan equine, the Eastern equine and the Western equine encephalitis group are rare and low (≥4 titer steps; Dobler, unpublished observations). Therefore we assume that cross reactions may mainly occur between Semliki Forest complex viruses like O′nyong nyong virus or Semliki Forest virus. Other non-African Semliki-Forest virus complex viruses, like Ross River virus or Mayaro virus do not seem to be responsible for the antibodies as the inhabitants of the areas tested did not leave the region. However, we cannot exclude that a so far unknown *Alphavirus* of the Semliki Forest virus complex is circulating and may cause infection with or without clinical symptoms. The question can only be answered by virus detection by isolation or molecular detection and characterization of genome parts.

This analysis of the seroprevalence for *Alphaviruses* adds to the picture of arthropod-borne infectious diseases in our study population. Together with previous reports on RVFV, rickettsiae of the typhus group and spotted fever group, we are demonstrating comparably high seroprevalences which could be caused by considerable exposure of the population to arthropod-borne infections other than malaria [23], [26], [27]. Akin to RVFV, a near-linear correlation of anti-*Alphavirus* IgG prevalence with age suggests endemic exposure rather than single or few epidemic events.

Acute *Alphavirus* infections such as chikungunya fever are neither known nor regularly diagnosed in the health facilities in the region, and might be overlooked by medical staff as a possible causative agent for febrile illness, leading to presentation at the health facility. Febrile disease in the area is mostly regarded as malaria by treating clinicians, despite the fact that our survey showed a marked reduction of *P. falciparum* infection since the introduction of artemether - lumefantrine as first line therapy in 2006 [28]. Therefore, the awareness for zoonoses as a possible underlying cause of febrile illness should be increased.

Our analysis shows that anti-*Alphavirus* IgG prevalence is associated with geographical features related to favourable mosquito breeding conditions. These include low to moderate elevations and flat terrain, which disposes to the formation of surface water collections [4].

Climate has been consistently pointed out as one of the major determinants for the distribution of vector borne diseases. Although lower larval rearing temperatures result in increased likelihood of adult female mosquitoes becoming infected with CHIKV or other arboviruses in laboratory experiments [29], [30], it is higher temperatures which are generally linked to more efficient disease transmission in laboratory and epidemiological investigations [31]. However, the temperature variables examined here dropped out of the multi-variable model due to lack of multi-variable significance, with the elevation variable obviously producing a better fit than the satellite-measured land surface temperatures. In La Reunion, the spread of *Ae. albopictus* has been found to be limited to elevations <1200 m in summer [32]; in Gharwal/India, spread was limited to <1.400 m. This corresponds well with the drop in seroprevalence in strata above 1197 m (table 1). We still assume that temperature is the causal limiting factor in higher elevations, not other elevation-dependent factors such as radiation or atmospheric pressure. It should thus be kept in mind that our elevation results should not be generalized to predict infection risk in other climatic settings.

Comparisons with our data on spread of other mosquito-borne infections, namely RVF, *Flaviviridae* and *P. falciparum* malaria, and the tick-borne spotted fever group (SFG) rickettsiae, produce interesting findings. The uni-variable association of *P. falciparum* to anti-*Alphavirus* IgG disappears when adjusting for age, elevation and slope, suggesting that this association was due to factors supporting the breeding of the different mosquito vectors alike. We did not test to distinguish between anti-CHIKV IgG and o′nyong′nyong virus IgG due to lack of capacities to perform the neutralization test, so it is possible that the seroprevalence is caused by more than one virus.

A similar association exists between bednet ownership and anti-*Alphavirus* IgG. Bednet ownership is not homogenous over the study area, but more frequent in areas of higher malaria transmission, which in our data are characterised by low elevation and even terrain, favouring standing surface water as mosquito breeding grounds. This not only supports *Anopheles* but also other mosquito species, so the use of bednets can be seen as a proxy for general abundance of mosquitoes – hence the positive association in uni-variable analysis. When corrected for elevation and slope of terrain, the association with bednets disappeared – showing that in malaria endemic areas, bednet ownership neither favours nor protects against *Alphavirus* infection. This may point towards a diurnally active vector such as *Aedes* spp., against which bednets do not protect.

The association of anti-*Alphavirus* IgG with RVFV and *Flavivirus* IgG, viruses sharing *Aedes* spp. as vector, is however retained in multivariable analysis. This shows that in addition to age, elevation and slope, additional relevant factors still influence the spread of these *Aedes* – borne infections which remain to be identified. Others have found higher seroprevalences in Cameroonians living under corrugated iron roofs vs. thatched grass roofs; furthermore, living in rural areas was associated with higher seroprevalences [33].

SFG rickettsiae and *Alphaviruses* are transmitted by completely different vectors (cattle ticks and mosquitoes respectively), therefore the reason for the observed association is not clear. Rural living conditions, defined by low population density and long distances to roads, was a risk factor in our analysis of SFG rickettsia IgG [27]. Others authors also found this to be a risk factor for anti-*Alphavirus* IgG [33], so it is possible that rural conditions are the factor which increases the risk for both of these diseases which do not have much else in common.

Interesting are the differences in geographical spread of anti-*Alphavirus* IgG versus RVFV IgG in our population. Anti-*Alphavirus* IgG is more evenly distributed in the two Kyela sub-sites, and is also common in other sites. RVFV IgG on the other hand concentrates along the shore of Lake Malawi and nearby watercourses. The preferred occurrence of RVFV along water bodies has been demonstrated in other settings as well [34], but does not seem to apply to anti-*Alphavirus* IgG positivity in our setting. This observation may imply that the vectors of RVFV in Kyela region are floodwater mosquito species and therefore need the shore of Lake Malawi, whilst the vectors of the *Alphavirus* may show an anthropophilic behaviour. The strong affinity to water, which applies for RVFV, but not for the *Alphavirus*, may also be related to a higher density of cattle as RVFV reservoir hosts along the water, or with the transovarial transmission of RVFV in diapausing *Aedes* mosquitoes along waterbodies [35]. It is also possible that RVFV requires a temperature optimum as suggested by our previous work, with a direct correlation with higher minimum temperatures, lower maximum temperatures, and positive influence of dense vegetation, conditions that are fulfilled mainly at the lakeshore [23]. The causative *Alphavirus*, despite probably limited to a smaller number of vector species compared to RVFV, seems to be less specific in terms of ecological conditions and seems to show a more anthropophilic behaviour, leading to a wider spread of the virus throughout the study area.

In summary, our data suggest that CHIKV or a closely related *Alphavirus* like o′nyong′nyong virus is circulating in the study area. If this virus causes disease, it could be an important cause of febrile illnesses in the region, and may be currently underdiagnosed. The linear relation of seropositivity to age suggests endemic rather than epidemic cycling, opposed to a study from Kenya where seropositivity was linked by the authors to epidemic exposure [14]. Kenya was reportedly affected by past CHIKV outbreaks, while there are no reports of outbreaks from our study area. A study from northern Tanzania reported acute CHIKV infection in 7.9% of febrile hospitalized patients, demonstrating that CHIKV circulates between epidemics in the country and may well be responsible for the seroprevalences in our study [13].

The power of our statements is further limited by the stratified nature of our study cohort, which results in prevalence levels slightly different from the general population. Further, the serological method used does not allow distinguishing between different *Alphavirus* species, and only gives information on cumulative lifetime infection risk. Therefore, prospective studies are needed to establish the rate of acute fever caused by CHIKV or other *Alphaviruses* in febrile patients. If the infecting *Alphavirus* is shown to be CHIKV or another *Alphavirus* of human medical importance these results should lead to a re-assessment of the local diagnostic algorithm for febrile illnesses, to take into account the endemic presence of the causative *Alphavirus(es)* in the area. These studies would also have to answer the question whether endemic strains do have a reduced pathogenicity, and have evaded detection by not causing the typical symptoms.

## Supporting Information

Strobe Checklist S1 The STROBE checklist for quality assurance in reporting of observational studies is attached.
(PDF)
